# A Case of Ruptured Exophytic Uterine Artery Pseudoaneurysm without Specific Risk Factors That Manifested Seven Days after Vaginal Delivery

**DOI:** 10.1155/2023/1637463

**Published:** 2023-11-25

**Authors:** Masatake Toshimitsu, Takayuki Iriyama, Jiro Sato, Osamu Abe, Mari Ichinose, Seisuke Sayama, Takahiro Seyama, Kenbun Sone, Keiichi Kumasawa, Yutaka Osuga

**Affiliations:** ^1^Department of Obstetrics and Gynecology, Faculty of Medicine, The University of Tokyo, 7-3-1 Hongo, Bunkyo-ku, Tokyo 113-8655, Japan; ^2^Department of Radiology, Tokyo Metropolitan Police Hospital, 4-22-1, Nakano, Nakano-ku, Tokyo 164-8541, Japan; ^3^Department of Radiology, Graduate School of Medicine, The University of Tokyo, 7-3-1 Hongo, Bunkyo-ku, Tokyo 113-8655, Japan

## Abstract

A uterine artery pseudoaneurysm (UAP) is a life-threatening complication during pregnancy and postpartum. Early diagnosis of exophytic UAP rupture is difficult due to the absence of vaginal bleeding. This study reports the case of a 31-year-old postpartum woman who presented with abdominal pain and fever seven days after vaginal delivery, without symptoms of maternal shock. Ultrasonography revealed a ruptured exophytic UAP with hemoperitoneum, which was confirmed using computed tomography. Interventional radiology confirmed that the site of the pseudoaneurysm was at the level of the uterine artery bifurcation, and embolization was performed immediately after diagnosis using a coil and n-butyl-2-cyanoacrylate. The patient's symptoms were relieved, and she was discharged 12 days after the embolization. At eight months postpartum, the UAP was not visible on transvaginal ultrasonography. Exophytic UAP can occur even in the absence of specific risk factors such as cesarean section or endometriosis, and the UAP may not necessarily rupture immediately after delivery. Obstetricians must remain aware of the possibility of exophytic UAP rupture manifesting as abdominal pain with postpartum fever, rather than as unstable vital signs. This is the first report of an exophytic UAP that occurred at the level of the uterine artery bifurcation. Identification of the sites where exophytic UAP can occur can aid in the early diagnosis of the condition.

## 1. Introduction

A uterine artery pseudoaneurysm (UAP) is primarily caused by injury to the uterine artery wall after a cesarean section, abortion, cervical conization, endometriosis, or myomectomy [1–4]. In true aneurysms, all three layers of the arterial wall form a bulge; in pseudoaneurysms, one or more layers of the arterial wall have a defect that forms a sac that communicates with the arterial lumen and is more likely to rupture than a true aneurysm. Therefore, a UAP is a potentially life-threatening condition. The clinical features of UAPs are dependent on the location of the pseudoaneurysm, which is categorized as inside or outside the uterus [5]. A UAP most commonly presents as asymptomatic or postpartum hemorrhage with vaginal bleeding when it communicates with the uterine cavity. However, when a UAP develops outside of the uterus, it may be associated with hydronephrosis due to ureteral obstruction and hemoperitoneum [6–9].

Although rare, the risk of developing an exophytic UAP outside of the uterus increases during pregnancy and after normal vaginal delivery, even in the absence of specific risk factors, such as hereditary connective tissue disorders and obstetric or gynecological surgery [8–11]. Due to the paucity of reports regarding exophytic UAPs forming outside of the uterus, their clinical features and management methods have not been established, leading to poor perinatal outcomes [8–11].

This report presents a case of a ruptured exophytic UAP associated with postpartum fever without maternal shock after vaginal delivery. The UAP had characteristic imaging features that can be used to elucidate its pathogenesis.

## 2. Case Presentation

A 31-year-old postpartum woman, G1 TPAL1001, was transferred to the University of Tokyo Hospital (Tokyo, Japan) eight days after delivery with a complaint of worsening abdominal pain. She had no history of vascular disease and no relevant previous medical, gynecological, or surgical histories. At the referring hospital, the patient had an uneventful pregnancy and delivered vaginally at 39 weeks and 2 days of gestation. She underwent augmentation with oxytocin and uterine fundal pressure due to arrested labor. The duration of the first and second stages of labor was 11 h 20 min and 48 min, respectively. The total volume of blood loss was 200 mL. The infant's birth weight was 3710 g, with an Apgar score of 1 at 1 min and 5 at 5 min. The pH of the umbilical artery was 7.08. The patient was discharged without further complications. On the seventh day postpartum, the patient experienced persistent abdominal pain and fever. The patient received antibiotic treatment with imipenem-cilastatin and isepamicin sulfate at the referral hospital. On the eighth day postpartum, she was transferred to the tertiary perinatal center at the University of Tokyo Hospital as her abdominal pain had not improved.

**Figure 1:**
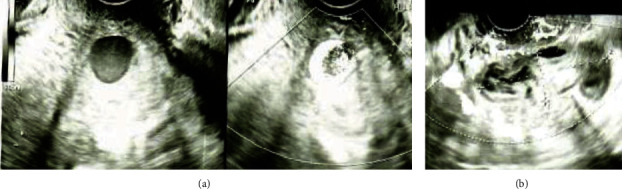
Transvaginal ultrasonographic images. (a) The left image shows the B-mode scan, and the right image shows color Doppler scan. To the left of the uterus, a 1.7 × 1.6 cm low-echoic mass is observed with turbulent flow inside it. (b) An 8 cm sized hematoma is observed in the pelvis.

**Figure 2:**
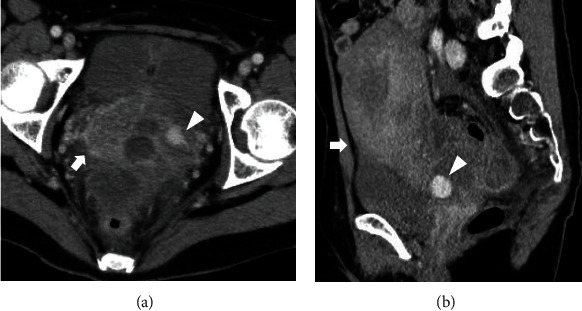
Contrast-enhanced computed tomographic images: (a) axial image and (b) sagittal image. The pseudoaneurysm, which originates from the left uterine artery, is outside of the uterine cavity. Hemoperitoneum is also observed. The arrowhead indicates the pseudoaneurysm. The arrow indicates the uterus.

**Figure 3:**
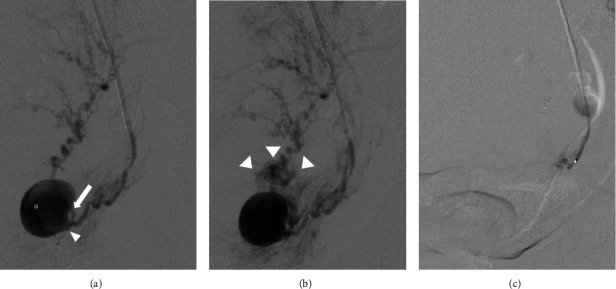
Angiographic images. (a) An angiogram reveals a pseudoaneurysm located at the level of the left uterine artery bifurcation. The asterisk indicates the uterine artery pseudoaneurysm. The arrow indicates the ascending branch. The arrowhead indicates the descending branch. (b) The uterine artery pseudoaneurysm with contrast extravasation is shown. The arrowheads indicate contrast extravasation. (c) Postembolization is completed with n-butyl-2-cyanoacrylate and coils.

**Figure 4:**
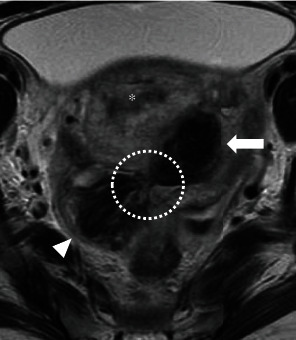
Magnetic resonance image on day two after embolization. Axial T2-weighted magnetic resonance image shows a hematoma within the broad ligament (arrow) adjacent to the postpartum uterus (asterisk) and hemoperitoneum (arrowhead). A continuous hematoma is observed from the thrombosed uterine artery pseudoaneurysm to the abdominal cavity, suggesting laceration of the left broad ligament (dotted circle).

On admission, the patient was hemodynamically stable and had no vaginal bleeding. Laboratory tests revealed a white blood cell count of 11.7 × 10^3^/*μ*L, C-reactive protein level of 14.8 mg/dL, and hemoglobin level of 9.4 g/dL. The patient's vaginal and blood cultures were negative. Transvaginal ultrasonography revealed a 17.6 × 16.7 mm hypoechoic mass adjacent to the left cervicocorporeal junction within an 8 cm hematoma (Figure 1). The mass had a turbulent swirling flow inside it, suggestive of an exophytic UAP with hemoperitoneum (Figure 1). Contrast-enhanced computed tomography showed a pseudoaneurysm outside of the uterine cavity that originated from the left uterine artery with contrast extravasation and hemoperitoneum, confirming the diagnosis of a ruptured exophytic UAP (Figure 2). Selective left iliac angiography performed via the right femoral artery revealed a pseudoaneurysm located at the level of the left uterine artery bifurcation into the ascending and descending branches with contrast extravasation (Figure 3). Embolization was achieved using n-butyl-2-cyanoacrylate (NBCA) and coils (Figure 3). The patient's abdominal pain resolved immediately after embolization. Follow-up ultrasonography revealed a thrombosed pseudoaneurysm with no flow and no exacerbation of the hemoperitoneum. Two days after embolization, magnetic resonance imaging revealed a continuous hematoma from the thrombosed UAP to the abdominal cavity, suggesting a laceration on the leaflet of the left broad ligament (Figure 4). There was no evidence of uterine rupture or abnormality. No clinical features of hereditary connective tissue disease were observed on physical or radiographic examination. The hemoperitoneum gradually subsided on subsequent ultrasonography. As infection could not be eliminated as the cause of the fever, tazobactam/piperacillin was administered at a dose of 13.5 g/day for ten days for possible sepsis, and the patient's fever subsided. The patient was discharged 12 days after the embolization. A postembolization follow-up eight months postpartum was uneventful, and the UAP was not visible on transvaginal ultrasonography.

## 3. Discussion

A case of a ruptured exophytic UAP at the level of the uterine artery bifurcation in a postpartum woman without specific risk factors is presented in this report. The patient was successfully treated via early interventional radiology. In this patient, postpartum fever without maternal shock was associated with a ruptured exophytic UAP. In addition, the outward formation of the UAP at the level of the uterine artery bifurcation was a distinctive imaging finding that was successfully treated using interventional radiology.

Pregnancy- and postpartum-related physiological changes that involve hemodynamics and hormones, such as an increase in estrogen levels during pregnancy, may affect aortic wall vulnerability [12, 13]. Even in the absence of hereditary connective tissue diseases such as Ehlers–Danlos syndrome and Marfan syndrome, the risk of a UAP forming outside of the uterus, spontaneous rupture of the uterine artery, or aortic dissection is increased during pregnancy and the postpartum period [10–12, 14]. In the current patient, the exact etiology of UAP is unknown. One possible mechanism for UAP development involves the combination of physiological changes during pregnancy and the postpartum period, as well as intra-abdominal pressure during labor. Parity, precipitated labor, the duration of the second stage of labor, and neonatal weight have also been identified as factors associated with obstetric injury [15, 16]. The current patient was at risk for obstetric injury due to the rapid second stage of labor (48 min), the fetal size (3710 g), and nulliparity, which may have prompted stretch stress to the uterine artery. The UAP may also have occurred during pregnancy. The patient's UAP was diagnosed after she experienced abdominal pain with fever on postpartum day seven. Therefore, a preceding infection may have contributed to the formation of the UAP. Uterine fundal pressure during the second stage of labor assists pregnant women in achieving vaginal delivery [17]; however, it is associated with maternal complications, including pelvic floor dysfunction, cervical laceration, perineal laceration, uterine rupture, and atonic bleeding [17]. The uterine fundal pressure may have augmented the intra-abdominal pressure and contributed to the development of the UAP via expulsion of the fetus; however, there is no evidence regarding an association between uterine fundal pressure and UAP formation. Based on numerous studies regarding maternal complications related to uterine fundal pressure, it is important to consider under what conditions its application is appropriate [18].

The main symptoms of exophytic UAP include abdominal pain and hypotension without vaginal bleeding [7, 9]. The current report is the first regarding a postpartum fever without hypotension. While the patient's blood and vaginal cultures were negative, these were obtained after antibiotic treatment was initiated at the referring hospital. Therefore, an infection prior to the UAP may have caused the patient's fever. The infection may also have been secondary to hemoperitoneum. The use of first-line antibiotics such as tazobactam/piperacillin has been suggested in cases of possible sepsis [19]. Microbiological culture work-up must be initiated prior to antibiotic treatment, and low ceiling antibiotics should be used based on regular sensitivity. However, there is a need for microbiologist guide in the use of high ceiling antibiotics which should never serve as first-line therapy.

A patient's hemodynamics are dependent on the rate and amount of blood loss due to ruptured UAP. The exophytic UAP did not rupture immediately postpartum in the current patient (postpartum day seven) or in a previously reported patient (postpartum day five) [9]. Therefore, the early diagnosis of UAP is difficult due to the variability of its clinical presentation and the interval between delivery and the onset of rupture. As UAP can lead to life-threatening hemorrhage, even if vascular events occur rarely, obstetricians must clinically check for and accurately diagnose UAP throughout the postpartum period, as in the present case.

A characteristic radiologic finding in this patient was the site of the exophytic UAP, which formed at the level of the uterine artery bifurcation. Although there is no evidence regarding the typical site of exophytic UAPs, the location of the present UAP suggests that the uterine artery bifurcation is an area vulnerable to mechanical force. This contributes to the elucidation of the pathogenesis of exophytic UAPs without specific risk factors. Furthermore, in this patient, the ipsilateral broad ligament had ruptured. The onset of abdominal pain seven days after delivery suggests that laceration of the broad ligament was caused by the postpartum UAP rupture, rather than during vaginal delivery.

Treatment options for UAP have advanced from open surgical management to interventional radiology, which can avoid the potential complications of laparotomy [20, 21]. Transarterial embolization is an effective therapeutic modality for UAP [20, 21]. The embolic materials vary; coils are the embolization material of choice to reduce the risk of recurrent bleeding in patients with exophytic UAP [7, 9]. The current patient was successfully treated via interventional radiology, with embolization using coils and NBCA. When the patient is hemodynamically stable, imaging studies may help identify the cause of hemoperitoneum, such as UAP, uterine rupture, or rupture of utero-ovarian vessels, and assisting with an accurate diagnosis and management.

In conclusion, postpartum fever and abdominal pain are strongly suggestive of a ruptured exophytic UAP in postpartum women. Importantly, an exophytic UAP can occur without specific risk factors and can rupture during the short-term postpartum period. Obstetricians must be aware of the possibility of exophytic UAP rupture and its occurrence at the level of the uterine artery bifurcation, as such awareness can lead to an early diagnosis and management of the UAP.

## Data Availability

All information and clinical images relevant to this case report are included in the article.
